# Modulating Electronic States of Cu in Metal‐Organic Frameworks for Emerging Controllable CH_4_/C_2_H_4_ Selectivity in CO_2_ Electroreduction

**DOI:** 10.1002/advs.202404931

**Published:** 2024-07-08

**Authors:** Mingxu Sun, Jiamin Cheng, Akihiko Anzai, Hirokazu Kobayashi, Miho Yamauchi

**Affiliations:** ^1^ Institute for Materials Chemistry and Engineering (IMCE) Kyushu University Motooka 744, Nishi‐ku Fukuoka 819‐0395 Japan; ^2^ Research Center for Negative Emissions Technologies (K‐NETs) Kyushu University Motooka 744, Nishi‐ku Fukuoka 819‐0395 Japan; ^3^ International Institute for Carbon‐Neutral Energy Research (WPI‐I2CNER) Kyushu University Motooka 744, Nishi‐ku Fukuoka 819‐0395 Japan; ^4^ Advanced Institute for Materials Research (WPI‐AIMR) Tohoku University 2‐1‐1 Katahira, Aoba‐ku Sendai 980–8577 Japan

**Keywords:** CO_2_ reduction, controllable selectivity, in situ spectroscopy, metal organic frameworks, UiO‐66

## Abstract

The intensive study of electrochemical CO_2_ reduction reaction (CO_2_RR) has resulted in numerous highly selective catalysts, however, most of these still exhibit uncontrollable selectivity. Here, it is reported for the first time the controllable CH_4_/C_2_H_4_ selectivity by modulating the electronic states of Cu incorporated in metal‐organic frameworks with different functional ligands, achieving a Faradaic efficiency of 58% for CH_4_ on Cu‐incorporated UiO‐66‐H (Ce) composite catalysts, **Cu/UiO‐66‐H (Ce)** and that of 44% for C_2_H_4_ on **Cu/UiO‐66‐F (Ce)**. In situ measurements of Raman and X‐ray absorption spectra revealed that the electron‐withdrawing ability of the ligand side group controls the product selectivity on MOFs through the modulation of the electronic states of Cu. This work opens new prospects for the development of MOFs as a platform for the tailored tuning of selectivity in CO_2_RR.

## Graphical abstract

The electrocatalytic CO_2_ reduction reaction selectivity is controlled through modulating the electronic structure of Cu in metal‐organic frameworks. Ligand‐altered UiO‐66 led to a notable shift from CH_4_ to C_2_H_4_, achieving high Faradaic efficiencies of 58% for CH_4_ and 44% for C_2_H_4_.

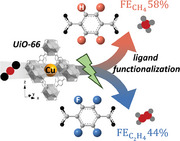

## 1 Introduction

The electrocatalytic CO_2_ reduction reaction (CO_2_RR) is considered to be a promising approach for the conversion of CO_2_ into fossil fuels.^[^ ^1^ ^]^ Copper (Cu), as one of the most efficient catalysts, can facilitate the conversion of CO_2_ into a range of products beyond CO,^[^ ^2^ ^]^ such as methane, ethylene, ethanol, and more.^[^ ^3^ ^]^ However, achieving precise control over the selectivity toward the target products within this range beyond CO remains a significant challenge.^[^ ^4^ ^]^ In the past decade, metal‐organic frameworks (MOFs) have received considerable attention as emerging CO_2_RR electrocatalysts due to their highly designable structure,^[^ ^5^ ^]^ and the Cu‐incorporated MOF composites have successfully produced various products including HCOOH,^[^ ^6^ ^]^ CO,^[^ ^7^ ^]^ CH_4_,^[^ ^8^ ^]^ CH_3_OH,^[^ ^9^ ^]^ C_2_H_4_,^[^ ^10^ ^]^ and C_2_H_5_OH.^[^ ^9^ ^]^ However, the selectivity toward the target product is often unpredictable due to the diversity of microenvironments in MOFs influenced by metal ions/clusters as nodes and organic ligands as linkers.^[^ ^11^ ^]^ A notable example is the use of Cu‐loaded ZIF‐8 in three similar studies as a precursor to obtain Cu single atom catalysts; however, these studies showed distinctly different selectivity for CH_3_OH,^[^ ^12^ ^]^ C_2_H_5_OH,^[^ ^13^ ^]^ and CH_3_OCH_3_,^[^ ^14^ ^]^ respectively. Herein, we first demonstrate the selectivity control of Cu incorporated in MOF composites by modulating the microscopic chemical environment around Cu sites, such as local pH and electron donativity, in conjunction with metal cluster nodes and functionalized organic ligands for the systematic study of selectivity in CO_2_RR.

**Scheme 1:**
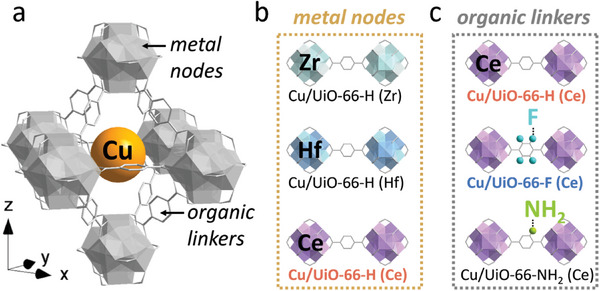
a) Structure illustration of Cu/UiO‐66‐L (M), H atoms are omitted for clarity. b) Zr, Hf, and Ce are selected as the metal nodes for Cu/UiO‐66‐H (M), respectively. c) H, F, and NH_2_ are selected as the functional groups of the organic linker for Cu/UiO‐66‐L (Ce), respectively.

We select UiO‐66 as a MOF support, which is composed of octahedral M_6_O_4_(OH)_4_ cluster nodes (M = Zr, Hf, Ce) as secondary building units connected by terephthalate ligand to form a porous 3‐D framework. The variable metal nodes and organic ligands in UiO‐66^[^ ^15^ ^]^ have offered a controllable local microenvironment that can selectively influence the catalytic performance of Cu.^[^ ^16^ ^]^ Furthermore, UiO‐66 is known for its high thermal and chemical stability,^[^ ^17^ ^]^ providing a useful platform to unravel the catalytic mechanism of Cu. We then propose a design of Cu‐incorporated UiO‐66 composite catalysts (Cu/UiO‐66‐L (M)). M and L represent metal nodes (M = Zr, Hf, Ce) and side groups of 1,4‐benzenedicarboxylic acid (BDC) linkers (L = H, F, NH_2_), respectively (**Scheme**. **1**). By choosing a suitable combination of a metal node and a ligand side group, we successfully suppressed the competitive HER from 84% to 14% and achieved variable selectivity for CH_4_ with Faradic efficiency ($FE_{CH_{4}}$) of 58% on **Cu/UiO‐66‐H (Ce)** and C_2_H_4_ with Faradic efficiency ($FE_{C_{2}H_{4}}$) of 44% on **Cu/UiO‐66‐F (Ce)**. In situ Raman and X‐ray absorption spectra of the UiO‐66 composites suggested that the selectivity between CH_4_ and H_2_ is influenced by variations in the local hydroxide (OH) concentration from differences in the metal nodes, and the selectivity switch from CH_4_ to C_2_H_4_ is attributed to the modulation of the surface electronic structure of active Cu sites by changing the side group of BDC ligand from ‐H to the ‐F with strong electron‐withdrawing ability.

## 2 Results and Discussion

We first consider the modulation of Cu sites through interaction with the different metal nodes of UiO‐66. Thus, we synthesized the Cu/UiO‐66‐H (M) (M = Zr, Hf, Ce) by solution infiltration of copper acetate in the presence of the pre‐synthesized corresponding UiO‐66 (Scheme 1a,b, see Supporting Information for synthesis details).^[^ ^16a^ ^]^ The powder X‐ray diffraction (PXRD) patterns and N_2_ sorption isotherms at 77 K for the UiO‐66‐H (M) (M = Zr, Hf, Ce) and the corresponding Cu/UiO‐66‐H (M) showed that the structure of all UiO‐66 is preserved after incorporation with Cu (Figures S1,S6, Supporting Information). The results of inductively coupled plasma atomic emission spectroscopy (ICP‐AES) showed that the loading amounts of Cu in Cu/UiO‐66‐H (M) (M = Zr, Hf, Ce) were 1.7, 2.7, and 4.0 wt.%, respectively (see Supporting Information for characterizations). In addition, the absence of diffraction peaks originating from Cu (Figure S1, Supporting Information) and the TEM images (Figures S2,S3, Supporting Information) for Cu/UiO‐66‐H (M) collectively suggested that Cu is atomically dispersed around UiO‐66.

**Figure 1:**
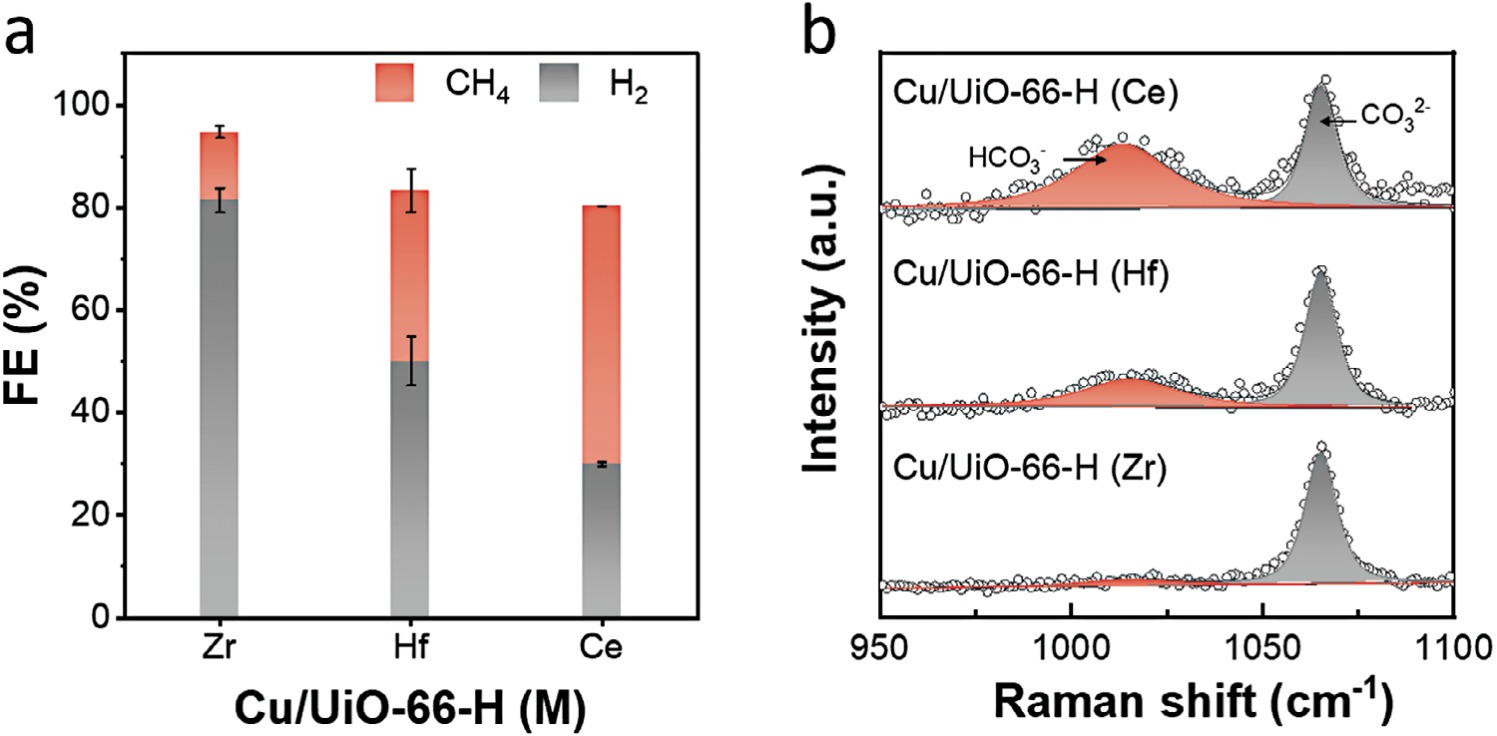
a) $FE_{CH_{4}}$ and $FE_{H_{2}}$ in CO_2_RR at −100 mA cm^−2^, and b) Raman spectra of v(HCO_3_^−^) and ν(CO_3_^2−^) at OCP by Cu/UiO‐66‐H (M).

We then evaluated the CO_2_RR performances of Cu/UiO‐66‐H (M = Zr, Hf, Ce) in a gas‐fed flow cell with 1 m KOH electrolyte at a current density (*j*) of −100 mA cm^−2^. **Figures** **1**a and S4 (Supporting Information) suggest that Cu/UiO‐66‐H (M) produced CH_4_ and H_2_ as main products; $FE_{CH_{4}}$ on **Cu/UiO‐66‐H (Zr)**, **Cu/UiO‐66‐H (Hf),** and **Cu/UiO‐66‐H (Ce)** were 13.3%, 33.3%, and 50.5%, respectively. To gain insight into the different selectivity for CH_4_ and H_2_, we investigated the chemical environment on Cu sites in Cu/UiO‐66‐H (M) by in situ Raman spectra measurements (see Supporting Information for measurement detail). The Raman spectra revealed two distinct bands for Cu/UiO‐66‐H (M) at ≈1015 and ≈1065 cm^−1^, corresponding to the HCO_3_^−^ stretching mode (*v*(HCO_3_^−^)) and CO_3_^2−^ stretching mode (*v*(CO_3_^2−^)), respectively (Figure 1b and Figure S5, Supporting Information). Considering the equilibrium of CO_3_^2−^ and HCO_3_^−^ under highly alkaline conditions (pH 14), where 99.98% of the CO_2_ derivative is CO_3_^2−^, which is calculated based on the Henderson‐Hasselbalch equation,^[^ ^18^ ^]^ the presence of distinguishable signals for *v*(HCO_3_^−^) suggests the occurrence of neutralization of local OH by the interaction with metal cluster nodes, and the extent of the neutralization depends on their metal ion. The metal nodes (M_6_O_4_(OH)_4_) of UiO‐66, containing µ_3_‐OH, M‐OH_2_, and M‐OH sites, act as Brønsted acid sites and can effectively influence the local OH concentration on Cu sites.^[^ ^19^ ^]^ The importance of the OH concentration on the catalyst surface in determining the direction of the pathway in CO_2_RR or HER has been widely discussed.^[^ ^4^, ^20^ ^]^ Among them, the relatively active acidic protons at the Ce nodes in UiO‐66 tend to be involved in the neutralization process via the reaction of CO_2_ and KOH (H^+^ + CO_2_ + OH^−^ → HCO_3_^−^), ultimately resulting in the generation of HCO_3_^–^. Similarly, these relatively active acidic protons on the Ce nodes can potentially interact with CO_2_RR intermediates, such as *CO hydrogenation, thereby promoting CH_4_ production. In summary, this discovery provides insights into the modulation of catalytic surface OH concentration to control the direction of CO_2_RR and HER.

Based on the effective HER suppression with the Ce metal node, we chose Ce as the metal node and further explored functions of the BDC ligand using Cu/UiO‐66‐L (Ce) (L = NH_2_, F, Scheme 1a,c). **Cu/UiO‐66‐F (Ce)** and **Cu/UiO‐66‐NH_2_ (Ce)** were synthesized by the same solution infiltration method as **Cu/UiO‐66‐H (Ce)** except for changing UiO‐66‐H (Ce) to UiO‐66‐F (Ce) and UiO‐66‐NH_2_ (Ce), respectively (see Supporting Information for synthesis details). The PXRD patterns of the obtained Cu/UiO‐66‐L (Ce) (L = NH_2_, F) were similar to those of the corresponding UiO‐66 analogs (Figure S1, Supporting Information), indicating that the structures of the UiO‐66 analogs were maintained after incorporation with Cu. Fourier transform infrared (FTIR) spectra confirmed the presence of ‐F and ‐NH_2_ functional groups in **Cu/UiO‐66‐F (Ce)** and **Cu/UiO‐66‐NH_2_ (Ce)**, respectively (Figure S7, Supporting Information). The TEM images of the Cu/UiO‐66‐L (Ce) (L = NH_2_, F) showed no observable particles associated with Cu, suggesting that Cu is atomically dispersed around the UiO‐66 (Figure S3, Supporting Information), which is also consistent with the results of Cu/UiO‐66‐H (M) (M = Zr, Hf, Ce). N_2_ sorption isotherms measured at 77 K for **Cu/UiO‐66‐F (Ce)** and **Cu/UiO‐66‐NH_2_ (Ce)** revealed that their composites showed typical type‐I sorption behavior originating from the microporosity, as is the case with the pristine UiO‐66‐F (Ce) and UiO‐66‐NH_2_ (Figure S6, Supporting Information). From the results of ICP‐AES, the loading amounts of Cu into Cu/UiO‐66‐L (Ce) (L = NH_2_, F) were 0.7 and 1.3 wt.%, respectively.

**Figure 2:**
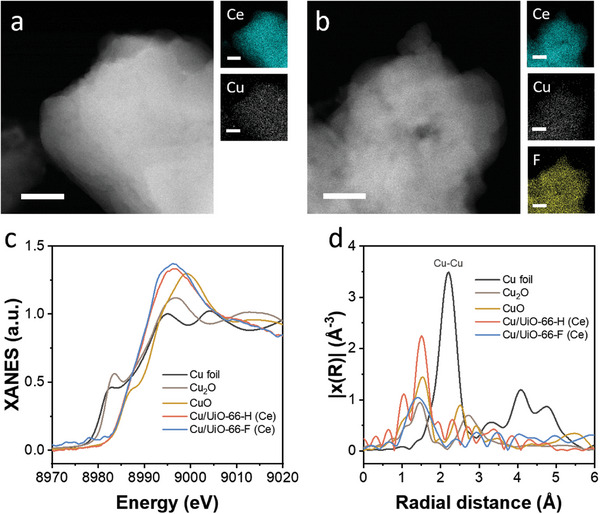
HAADF‐STEM image and EDX maps of a) **Cu/UiO‐66‐H (Ce)** and b) **Cu/UiO‐66‐F (Ce)** Scale bar = 100 nm. (The samples for HAADF‐STEM and EDX measurements were treated at a *j* of −10 mA cm^−2^ for 10 min. see Supporting Information of electrochemical measurements for details). c) Ex situ XANES and d) FTs of the k^2^‐weighted EXAFS oscillations of **Cu/UiO‐66‐H (Ce)** and **Cu/UiO‐66‐F (Ce)** with Cu foil, Cu_2_O and CuO as references at Cu K‐edge.

To further investigate the composite structure of **Cu/UiO‐66‐H (Ce)** and **Cu/UiO‐66‐F (Ce)**, we performed high‐angle annular dark‐field scanning transmission electron microscopy (HAADF‐STEM) and energy‐dispersive X‐ray (EDX) elemental mapping. As shown in **Figure** **2**b and Figures S8 and S9 (Supporting Information) the HAADF‐STEM images of **Cu/UiO‐66‐H (Ce)** and **Cu/UiO‐66‐F (Ce)** revealed the absence of particles associated with Cu. STEM‐EDX mappings of Cu, Ce, and F which are the constituent elements of **Cu/UiO‐66‐H (Ce)** and **Cu/UiO‐66‐F (Ce)**, demonstrated uniform distribution of Cu throughout the entirety of the corresponding UiO‐66 (Figure 2a,b). Similarly, HAADF‐STEM images and STEM‐EDX mappings of **Cu/UiO‐66‐NH_2_ (Ce)** showed similar results to those of **Cu/UiO‐66‐H (Ce)** and **Cu/UiO‐66‐F (Ce)** (Figure S10, Supporting Information). To gain further insight into the local structure and electronic states of Cu species within Cu/UiO‐66‐L (Ce), we performed X‐ray absorption spectroscopy (XAS) measurements. The Cu K‐edge X‐ray absorption near‐edge structure (XANES) spectra indicated that the absorption edge positions of Cu/UiO‐66‐L (Ce) are similar to those of CuO, suggesting a positive charge valence state of Cu species close to +2 (Figure 2c). Furthermore, the Fourier transforms (FTs) of the extended X‐ray absorption fine structure (EXAFS) oscillations showed main peaks at 1.5 Å for **Cu/UiO‐66‐H (Ce)** and **Cu/UiO‐66‐F (Ce)** (Figure 2d). Notably, the Cu─Cu peak ≈ 2.2 Å was absent in the cases of Cu/UiO‐66‐L (Ce), indicating that Cu species are atomically dispersed (Figure 2d). These results demonstrate that positively charged Cu species are homogeneously distributed in Cu/UiO‐66‐L (Ce).

**Figure 3:**
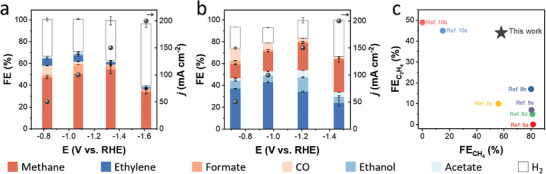
CO_2_RR performance of a) **Cu/UiO‐66‐H (Ce)** and b) **Cu/UiO‐66‐F (Ce)**. c) FE of CH_4_ and C_2_H_4_ compared with state‐of‐art MOF electrocatalysts.

We then examined the effect of the side groups of BDC on product selectivity. As shown in **Figure** **3**a, **Cu/UiO‐66‐H (Ce)** predominantly produced CH_4_ in the applied potential range of −0.83 to −1.61 V and achieved $FE_{CH_{4}}$ of 58% at −1.33 V. In contrast, **Cu/UiO‐66‐NH_2_ (Ce)** exhibited low $FE_{CH_{4}}$ of 12% at −1.4 V (Figure S13, Supporting Information), demonstrating that the BDC with −NH_2_ reduces the CH_4_ selectivity (Figures S11 and S12, Supporting Information). It should be noted that **Cu/UiO‐66‐F (Ce)** produced C_2_H_4_ as the main product in a similar applied potential range as **Cu/UiO‐66‐H (Ce)**, and $FE_{C_{2}H_{4}}$ was 44% at −0.96 V (Figure 3b). The change of the side group from –H to –F shifted the product selectivity from CH_4_ to C_2_H_4_, which provides a pioneering method to alter the main product on Cu in CO_2_RR by employing ligand functionalization in MOFs. The variable selectivity for CH_4_ and C_2_H_4_ in this work is comparable to those of state‐of‐the‐art MOF electrocatalysts, as illustrated in Figure 3c ^[^ ^8^, ^10^ ^]^ In addition, the results of the stability test indicated that **Cu/UiO‐66‐H (Ce)** and **Cu/UiO‐66‐F (Ce)** maintained *j* with high selectivity at –0.8 V (Figure S14, Supporting Information). In the XRD patterns of **Cu/UiO‐66‐H (Ce)** and **Cu/UiO‐66‐F (Ce)** after CO_2_RR (Figure S15, Supporting Information), although the crystallinity of corresponding UiO‐66 decreased, the diffraction peaks of Cu were not observed, and the atomically dispersed Cu species were retained. The STEM‐EDX of **Cu/UiO‐66‐H (Ce)** and **Cu/UiO‐66‐F (Ce)** after CO_2_RR also revealed that the homogeneous distributions of Cu and the constituent elements of corresponding UiO‐66 were maintained (Figures S16 and S17, Supporting Information).

**Figure 4:**
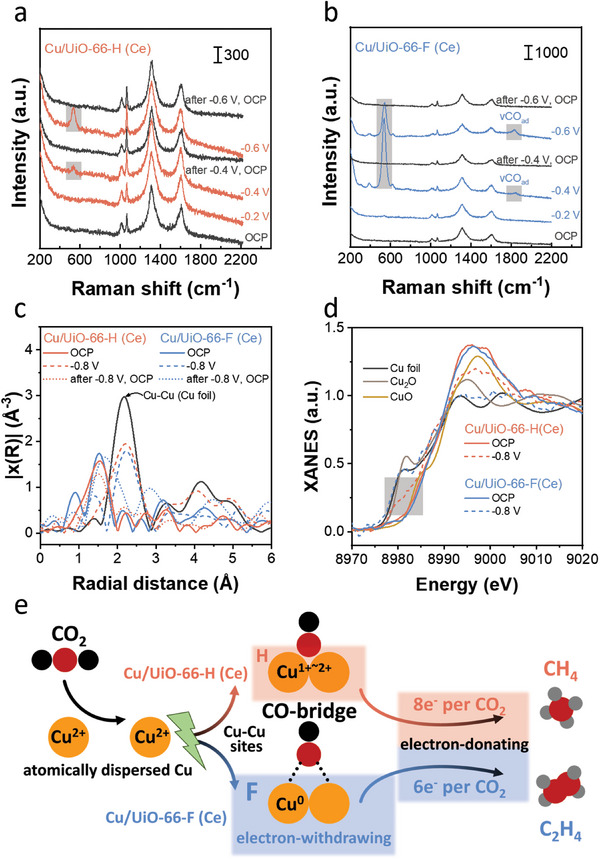
In situ Raman spectra of a) **Cu/UiO‐66‐H (Ce)** and b) **Cu/UiO‐66‐F (Ce)** recorded at OCP and various potentials. c) In situ FTs of the k^2^‐weighted Cu K‐edge EXAFS oscillations and d) XANES spectra of **Cu/UiO‐66‐H (Ce)** and **Cu/UiO‐66‐F (Ce)** recorded at OCP and −0.8 V versus RHE with Cu foil, Cu_2_O, and CuO as references at Cu K‐edge. e) Mechanistic description of CH_4_ and C_2_H_4_ formation for **Cu/UiO‐66‐H (Ce)** and **Cu/UiO‐66‐F (Ce)**, respectively.

To explore the origin for the shift in the main product from CH_4_ to C_2_H_4_ on Cu/UiO‐66 (Ce) by changing side group from –H to –F, we performed in situ Raman spectra measurements for **Cu/UiO‐66‐H (Ce)** and **Cu/UiO‐66‐F (Ce)**. Compared to the open circuit potential (OCP), a new distinct peak appeared at ≈535 cm^–1^ when the potential was applied to –0.4 V. This phenomena is attributed to the reduction of Ce^4+^ to Ce^3+^,^[^ ^21^ ^]^ forming the oxygen vacancies (**Figure** **4**a,b).^[^ ^22^ ^]^ It is noted that the peaks at ≈535 cm^−1^ for **Cu/UiO‐66‐F (Ce)** at –0.4 V were much stronger than those for **Cu/UiO‐66‐H (Ce)**. These results suggest that the valence state of Ce nodes in Cu/UiO‐66 (Ce) is reduced from Ce^4+^ to Ce^3+^ with different degrees, and the Ce in **Cu/UiO‐66‐F (Ce)** is closer to Ce^3+^ compared to **Cu/UiO‐66‐H (Ce)** during CO_2_RR. Interestingly, the peak at ≈535 cm^−1^ of **Cu/UiO‐66‐H (Ce)** and **Cu/UiO‐66‐F (Ce)** disappeared under the OCP after the application of potential, and the spectra returned to the initial state before the application of the potential. This reversible behavior of Raman spectra to potential application was also observed in the second cycle (Figure 4a,b). These results imply that both **Cu/UiO‐66‐H (Ce)** and **Cu/UiO‐66‐F (Ce)** possess specific electronic and geometric structures during CO_2_RR.

To further explore their feature structures during CO_2_RR, in situ XAS measurements were performed for **Cu/UiO‐66‐H (Ce)** and **Cu/UiO‐66‐F (Ce)** (Figure 4c,d). This is an effective method for elucidating the in situ feature structure of MOFs.^[^ ^23^ ^]^ FTs of the EXAFS spectra indicate that, at OCP, the local structure of Cu in both **Cu/UiO‐66‐H (Ce)** and **Cu/UiO‐66‐F (Ce)** closely resembles that observed under ambient conditions (Figure 2d). However, upon applying a potential of −0.8 V, the original peak observed at 1.5 Å at OCP disappeared, while a new peak emerged at 2.2 Å, attributed to Cu─Cu bonds (Figure 4c). This result indicates that the atomically dispersed Cu species undergo restructuring to form Cu─Cu active sites for CO_2_RR (Figure 4e). Furthermore, in the in situ Raman spectra of **Cu/UiO‐66‐F (Ce)**, under applied potentials of −0.4 and −0.6 V (Figure 4b), we observed *CO species adsorbed on the bridge sites (CO‐bridge) of between adjacent Cu atoms at 1835 cm^−1^.^[^ ^24^ ^]^ This CO‐bridge species has been discussed as an important intermediate for ethylene production.^[^ ^25^ ^]^ Therefore, these Cu─Cu atom pairs may serve as crucial sites for CO‐bridge adsorption during ethylene formation (Figure 4e). Considering the absence of aggregated Cu particles in the STEM‐EDX mappings (Figure S16, Supporting Information) and the existence of Cu‐Cu atom pairs from the in situ XAS and Raman spectra (Figure 4a–c), clustering of Cu atoms is considered to act as active sites during CO_2_RR. It should be noted that similar to the trend representing Ce valence state changes observed in situ Raman spectra (Figure 4a,b), the Cu─Cu bond also disappeared and returned to isolated Cu atom state at OCP after the application of potential (Figure 4c). These results further confirmed that both **Cu/UiO‐66‐H (Ce)** and **Cu/UiO‐66‐F (Ce)** exhibit distinct structures during CO_2_RR. In addition, the in situ XANES spectra indicate that the valence of Cu in **Cu/UiO‐66‐H (Ce)** decreased from near +2 to a range between +1 and +2 upon applying a potential of −0.8 V. In contrast, the valence of Cu in **Cu/UiO‐66‐F (Ce)** changed to a metallic state under the same condition (Figure 4d).

In brief, in situ Raman (Figure 4a,b) and XAS (Figure 4d) spectra collectively indicated that functionalization with −F promotes the reduction of Ce^4+^ to Ce^3+^ and Cu^2+^ to Cu^0^ in **Cu/UiO‐66‐F (Ce)** during CO_2_RR, compared to **Cu/UiO‐66‐H (Ce)**. These phenomena can be summarized as the strong electron‐withdrawing effect of ‐F on BDC aiding electron accumulation on **Cu/UiO‐66‐F (Ce)**, thereby facilitating the reduction of Ce^4+^ and Cu^2+^ in **Cu/UiO‐66‐F (Ce)**. Notably, the reduction of one equivalent of CO_2_ to CH_4_ requires the consumption of 8 electrons, while that to C_2_H_4_ only utilizes 6 electrons as illustrated in Figure 4e. Hence, the shift in selectivity from CH_4_ to C_2_H_4_ on **Cu/UiO‐66‐F (Ce)** is caused by the electron accumulation on the surface of **Cu/UiO‐66‐F (Ce)**, of which electron‐donating ability toward substrate molecules outside MOF is relatively low.

## 3 Conclusion

To conclude, we demonstrated for the first time the systematic control of selectivity in CO_2_RR by modulating the electronic states of Cu sites through functionalization of UiO‐66 MOFs. The ligand functionalization of UiO‐66 altered main product with high FEch_4_ of 58% for Cu/UiO‐66‐H (Ce) and FEc_2_ h_4_ of 44% for Cu/UiO‐66‐F (Ce).

In situ, Raman and X‐ray absorption spectra suggested that the controlled selectivity of Cu/UiO‐66‐L (Ce) by the ligand functionalization originates from the modulation of the surface electronic structure of active Cu sites by strong electron‐withdrawing of −F on BDC. This work gives valuable information on a design guideline to develop useful CO_2_RR catalysts by careful selection of the metal nodes and the functional group of MOFs.

## Conflict of Interest

The authors declare no conflict of interest.

## Supporting information

Supporting Information

## Data Availability

The data that support the findings of this study are available from the corresponding author upon reasonable request.
