# Supplementary material for: Modulating Electronic States of Cu in Metal‐Organic Frameworks for Emerging Controllable CH4/C2H4 Selectivity in CO2 Electroreduction
Source: Adv Sci (Weinh). 2024 Jul 8;11(34):2404931. doi: 10.1002/advs.202404931 (PMC11425631; doi:10.1002/advs.202404931)
Supplement: Supplementary file 1 — Supporting Information [file ADVS-11-2404931-s001.docx]

Supporting Information©Wiley-VCH 202169451 Weinheim, Germany

Modulating Electronic States of Cu in Metal-Organic Frameworks for Emerging Controllable CH_4_/C_2_H_4_ Selectivity in CO_2_ Electroreduction

Mingxu Sun,^✝[b]^ Jiamin Cheng,^✝[a][b]^ Akihiko Anzai,^[b]^ Hirokazu Kobayashi,*^[a]^ and Miho Yamauchi*^[a][b][c][d]^

**Abstract:** The intensive study of electrochemical CO_2_ reduction reaction (CO_2_RR) has resulted in numerous highly selective catalysts, however, most of these still exhibit uncontrollable selectivity. Here, we report for the first time the controllable CH_4_/C_2_H_4_ selectivity by modulating the electronic states of Cu incorporated in metal-organic frameworks with different functional ligands, achieving a Faradaic efficiency of 58% for CH_4_ on **Cu/UiO-66-H (Ce)** and that of 44% for C_2_H_4_ on **Cu/UiO-66-F (Ce)**. In-situ measurements of Raman and X-ray absorption spectra revealed that the electron-withdrawing ability of the ligand side group controls the product selectivity on MOFs through the modulation of the electronic states of Cu. This work opens new prospects for the development of MOFs as a platform for the tailored tuning of selectivity in CO_2_RR.

DOI: 10.1002/anie.2021XXXXX

Table of Contents

Experimental Procedures 3

Synthesis of UiO-66-H (Zr) 3

Synthesis of UiO-66-H (Hf) 3

Synthesis of UiO-66-H (Ce) 3

Synthesis of UiO-66-F (Ce) 3

Solvent-assisted linker exchange on UiO-66-H (Ce) to form UiO-66-NH_2_ (Ce) 4

Synthesis of Cu/UiO-66-L (M) 4

Preparation of gas diffusion electrode (GDE) 4

Characterizations 4

Electrochemical measurements 5

In-situ Raman spectroscopy measurements 5

In-situ X-ray absorption spectroscopy measurements 5

Results and Discussion 7

Figure S1 to Figure S16 7

# Author Contributions 23

Experimental Procedures

**Materials**

All chemicals used in this study were obtained from commercial sources (Tokyo Chemical Industry Co., Ltd., Tokyo, Japan) and were used without further purification, unless otherwise noted. Specifically, Solvents including *N,N*-dimethylformamide (DMF) and ethanol were purchased from FUJIFILM Wako Pure Chemical Corporation, Japan.

**Synthesis of UiO-66-H (Zr)**

Acetic acid (2.75 mL, 0.048 mol) and zirconium (IV) *n*-propoxide [Zr(OPr)_4_] (64.2 μL, 0.143 mmol) were added sequentially to a 20 mL scintillation vial containing terephthalic acid (25 mg, 0.150 mmol) dissolved in DMF (9.75 mL). The vial was sealed and placed in a 50^o^C isothermal oven overnight. UiO-66-H (Zr) was collected using a centrifuge (8,000 rpm, 5 min), immersed in DMF (5 mL × 3) over 24 h period and in acetone (5 mL × 3) over another 24 h period. Finally, UiO-66-H (Zr) was dried under dynamic vacuum overnight at 80^o^C.

**Synthesis of UiO-66-H (Hf)**

In a typical synthesis, DMF 30 ml was added to a mixture of the hafnium (IV) chloride (HfCl_4_) (550 mg, 1.7 mmol) and acetic acid (2.0 ml, 0.066 mol) additive and sonicated to dissolve, forming a slightly cloudy colorless solution. Next, the terephthalic acid (285 mg, 1.7 mmol) was added to the solution and again sonicated to dissolve. Finally, water (100 μL) was added, the solution was stirred, placed into a glass pressure vessel, sealed, and heated in a pre-heated oven at 120 °C for 24 hours. UiO-66-H (Hf) was collected using a centrifuge (8,000 rpm, 5 min), immersed in DMF (20 mL × 3) over 24 h period and in acetone (20 mL × 3) over another 24 h period. Finally, UiO-66-H (Hf) was dried under dynamic vacuum overnight at 80^o^C.

**Synthesis of UiO-66-H (Ce)**

Terephthalic acid (286 mg, 1.7 mmol) was dissolved in DMF (9.6 ml). Cerium ammonium nitrate [(NH_4_)_2_Ce(NO_3_)_6_] (939 mg, 1.7 mmol) was dissolved in H_2_O (3.2 ml) and the solution was dropped into the solution of terephthalic acid. Then, the mixture was sonicated and heated to 100^o^C under stirring for 20 min. The suspension thus produced was centrifuged, and the obtained pale-yellow solid was collected and washed three times with DMF and three times with ethanol. The solid was then dried under dynamic vacuum at 80^o^C for 24 h.

**Synthesis of UiO-66-F (Ce)**

Tetrafluoroterephthalic acid (406 mg, 1.7 mmol) was dissolved in DMF (9.6 ml). (NH_4_)_2_Ce(NO_3_)_6_ (939 mg, 1.7 mmol) was dissolved in H_2_O (3.2 ml) and the solution was dropped into the solution of tetrafluoroterephthalic acid. Then, the mixture was sonicated and heated to 100^o^C under stirring for 20 min. The suspension thus produced was centrifuged, and the obtained solid was collected and washed three times with DMF and three times with ethanol. The solid was then dried under dynamic vacuum at 80^o^C for 24 h.

**Solvent-assisted linker exchange on UiO-66-H (Ce) to form UiO-66-NH_2_ (Ce)**

UiO-66-H (Ce) (117 mg), 2-aminoterephthalic acid (260 mg), and 35 mL of methanol were vortexed to ensure dispersion in the solvent and then sonicated at room temperature for five minutes. The resulting solid was centrifuged and washed three times with DMF to remove any excess linkers, further washed three times with ethanol, and then soaked in ethanol overnight. The resulting powder was collected by centrifugation and dried in a vacuum oven at 80^o^C for half an hour to remove excess solvent. The powder product was transferred to a sample tube and then activated under vacuum at 40^o^C for 24 hours to remove any residual solvent molecules. The success of the linker exchange was probed by element analysis with exchange of 97% to form UiO-66-NH_2_ (Ce).

**Synthesis of Cu/UiO-66-L (M)**

UiO-66-L (M) (100 mg) was dispersed in 100 mL of ethanol and then Cu(OAc)_2_·H_2_O (39.3 mg, 0.2 mmol) was dissolved in the above suspension under constant stirring over night. Cu/UiO-66-L (M) was collected using a centrifuge (8,000 rpm, 5 min) and washed three times with ethanol. Finally, the obtained Cu/UiO-66-L (M) was dried under dynamic vacuum overnight at 80^o^C.

**Preparation of gas diffusion electrode (GDE)**

The catalyst Cu/UiO-66 ink was prepared by adding 1 mg catalyst into a mixed solution of 2 mL DMF and 2 mg polyvinylidene difluoride (PVDF), and then ultrasonically treated it for 5 minutes, and finally formed a mixed ink. Then, the GDE was prepared by filtering the ink on carbon paper with a catalyst loading density of ~0.25 mg cm^-2^ and dried.

**Characterizations**

Transmission electron microscopy (TEM) images were captured using a JEM-2100 HC instrument operated at 100 kV accelerating voltage. Scanning transmission electron microscopy (STEM) and energy dispersive X-ray spectroscopy (EDX) element mapping images were carried out on a JEM-ARM200F (JEOL Co., Tokyo, Japan) instrument operated at 200 kV accelerating voltage. Fourier transform infrared (FTIR) spectra were recorded using a Nicolet iS50 spectrometer. X-ray absorption fine structure (XAFS) spectra were collected at the BL06 beam line of Kyushu Synchrotron Light Research Center (SAGA-LS, Japan), using a silicon (111) double crystal monochromator. The data were processed by Athena and Artemis included in the Ifeffit package. Inductively coupled plasma mass spectrometry (ICP–MS) was performed with a Thermo Scientific iCAP6300 Radial spectrometer. As sample preparation for the inductively coupled plasma atomic emission spectroscopy (ICP-AES) measurement, pre-weighed samples were dissolved in 3:7 (v/v) solution of HNO_3_–HCl. From the results of ICP-AES measurements, the loading amounts of Cu in **Cu/UiO-66-H (Zr)**, **Cu/UiO-66-H (Hf)**, **Cu/UiO-66-H (Ce)**, **Cu/UiO-66-F (Ce)** and **Cu/UiO-66-NH2 (Ce)** were determined to be 1.7, 2.7, 4.0, 1.3 and 0.7 wt%, respectively. N_2_ sorption isotherms were collected at 77 K by a BELSORP-MAX (BEL Japan, Inc.) using volumetric techniques. The samples were activated over 12 h at 403 K under vacuum before the measurement. The structures of samples were investigated by powder X-ray diffraction (XRD) analysis using a Bruker D2-Phaser and PANalytical diffractometer. The element analysis of N in Cu/UiO-66-NH_2_ (Ce) was measured by JSL Micro Corder JM11.

**Electrochemical measurements**

Electrochemical measurements were conducted using a Parstat MC potentiostat (Princeton Applied Research) in a gas diffusion flow reactor. A prepared GDE acted as a cathode, which needs to be activated by *j* of 10 mA cm^-2^ before electrochemical measurements, Nafion 117, a Ni foam sheet, and Ag/AgCl (3.0 M NaCl) served as a membrane, anode, and reference electrode respectively, clamped together with gaskets. The electrolyte used was 1 M KOH (85%, Wako), circulated through the electrochemical cell with a peristaltic pump at 2.5 ml min^−1^. CO_2_ (99.99%) flow rate was controlled at 30 ml min^−1^. To calibrate the potential with respect to the reversible hydrogen electrode (RHE), we used this equation: *E* (vs. RHE) = *E* (vs. Ag/AgCl) + 0.209 V + 0.0592 × pH. This calibration allowed accurate potential measurements relative to the RHE. The 80% iR drop was compensated using electrochemical impedance spectroscopy (EIS) at open circuit potentials, immediately following each set of CO_2_RR experiments. Effluent gas products from the cathodic compartment were analyzed using gas chromatography (GC, Agilent 490), and liquid products were analyzed by high-performance liquid chromatography (HPLC, Shimadzu LC-20AD). The Faradaic efficiency of gas products (*FE*_gas_) and liquid products (*FE*_liquid_) has been determined using the following equations:

$${FE}_{\mathrm{gas}}=\frac{nFC_{i}vP}{QRT}\times100\%$$

(S1)

$${FE}_{\mathrm{liquid}}=\frac{nFC_{i}V}{Q}\times100\%$$

(S2),

where *n* represents the number of electrons transferred to produce one molecule of the specific product *i*. *F* is the Faraday constant. *C_i_* stands for the concentration of product *i*, as determined through GC or HPLC analysis. *v* denotes the flow rate of CO_2_. *P*, *T*, and *Q* represent pressure, temperature, and total charge observed during the experimental process. *R* is the gas constant. *V* represents the volume of the electrolyte.

**In-situ Raman spectroscopy measurements**

Raman spectra were acquired using a Renishaw Raman spectrometer equipped with a 785 nm excitation laser and a 1200 mm^-1^ grating. The laser operated at a power of 200 µW, and observations were conducted using a 50× magnification objective. The recorded spectra spanned the 200–2300 cm^-1^ range, with each spectrum captured over a 10 second exposure time. Prior to collecting each Raman spectrum, CO_2_RR was pre-stabilized for 120 seconds, ensuring stable reaction conditions for data acquisition. A custom-made electrochemical Raman flow cell was employed. This flow cell design featured a thin electrolyte layer of approximately 3 mm, enabling direct laser irradiation of the catalyst surface through a light window. Within this setup, a prepared GDE served as the working electrode, which needs to be activated by *j* of 10 mA cm^-2^ before CO_2_RR. The counter electrode was a coiled Pt wire (100 × 0.5 mm^2^), and Ag/AgCl (3.0 M NaCl) was utilized as the reference electrode. Nafion-117 was strategically positioned to separate the catholyte and anolyte chambers. The electrolyte was a 1 M KOH, flowing at a rate of 2.5 mL min^-1^. Simultaneously, CO_2_ was continuously introduced into the gas chamber at a controlled flow rate of 50 mL min^-1^ throughout the test. The increase in local OH⁻ concentration during CO_2_RR and/or HER influences the formation of pH sensitive species such as CO_3_^2−^ and HCO_3_^−^. Therefore, to investigate the effect of metal cluster nodes on OH⁻, Raman spectroscopy was measured under open circuit potential for Figure 1b and S5.

**In-situ X-ray absorption spectroscopy measurements**

In-situ X-ray absorption fine structure (XAFS) experiments were performed under the same conditions as the electrochemical tests, using a modified flow cell with an opening in the gas chamber sealed by Kapton tape. In-situ Cu K-edge XAFS spectra were measured at the Kyushu University beamline BL06 of Kyushu Synchrotron Light Research Center (SAGA-LS, Japan) with an electron storage ring operating at the energy of 1.4 GeV. The energy range of this light source (bending magnet) is 2.1–23 keV. A silicon (111) double-crystal monochromator was used to collect the incident X-ray beam. The typical photon flux is 1010 photons per second. In-situ XAFS spectra were recorded in the fluorescence mode using a four-element silicon drift detector (SDD). The reference spectra of CuO, Cu_2_O and Cu were recorded in the transmission mode. The powders of the reference samples were well mixed with boron nitride (BN) and then pressed into a pellet for measurement. Data processing was performed using Athena and Artemis, which are included in the Ifeffit package.1

Results and Discussion


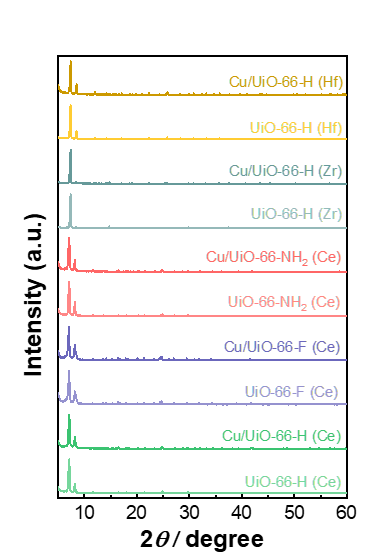


**Figure S1.** PXRD patterns of UiO-66-L (M) and Cu/UiO-66-L (M).


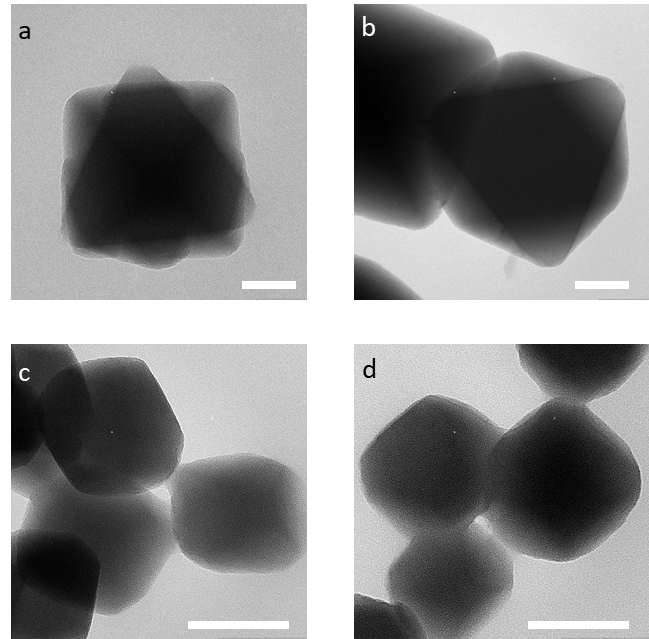


**Figure S2.** TEM images of a) UiO-66-H (Zr), b) Cu/UiO-66-H (Zr), c) UiO-66-H (Hf), and d) Cu/UiO-66-H (Hf). Scale bar = 100 nm.


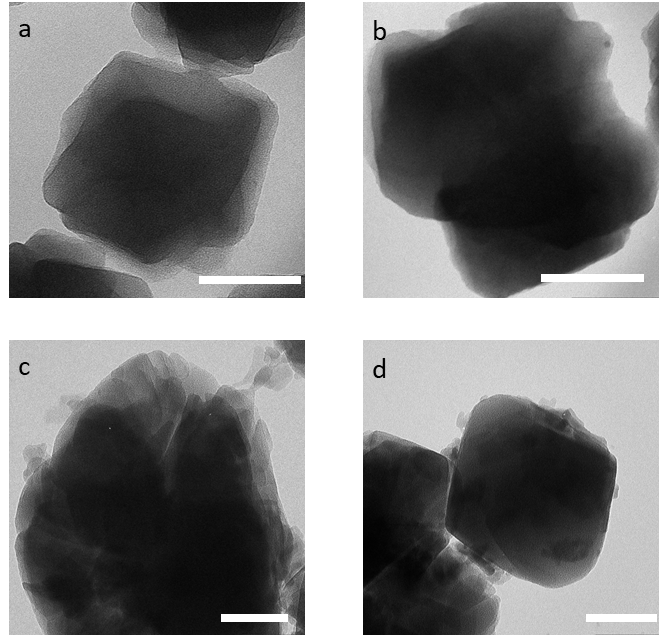


**Figure S3.** TEM images of a) UiO-66-H (Ce), b) Cu/UiO-66-H (Ce), c) UiO-66-F (Ce), and d) Cu/UiO-66-F (Ce). Scale bar = 100 nm.


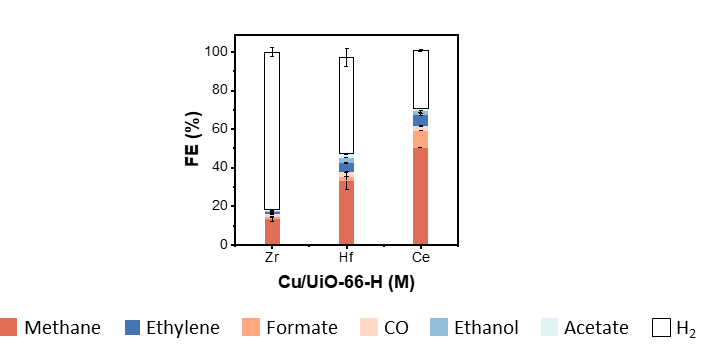


**Figure S4.** CO_2_RR performance of Cu/UiO-66-H (M).

**Figure S5.** a) Raman spectra of Cu/UiO-66-H (M) at OCP.


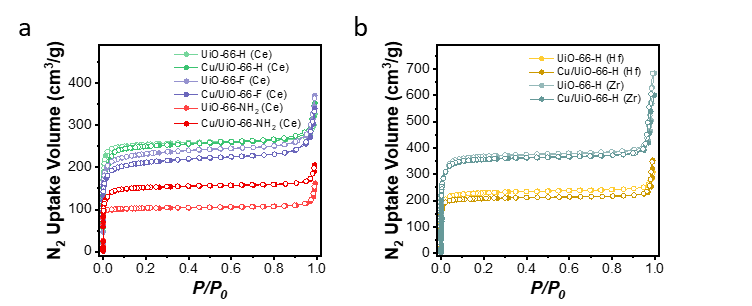


**Figure S6.** a), b) N_2_ sorption isotherms of UiO-66-L (M) and Cu/UiO-66-L (M).


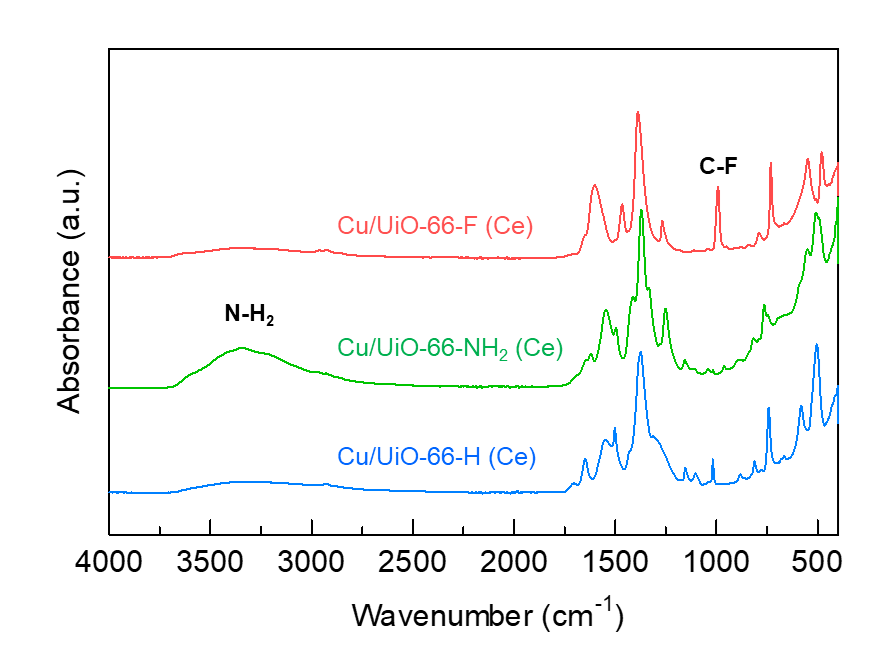


**Figure S7.** FTIR of Cu/UiO-66-H (Ce), Cu/UiO-66-F and (Ce)Cu/UiO-66-NH_2_ (Ce).


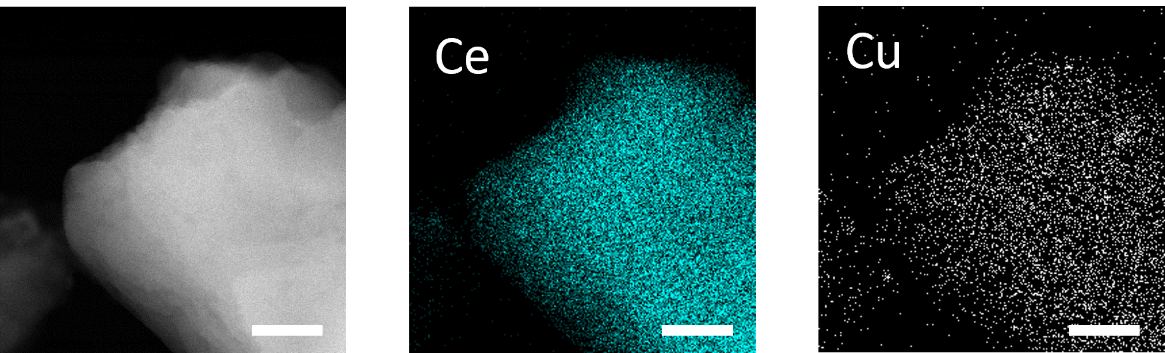


**Figure S8.** HAADF-STEM image and EDX maps of Cu/UiO-66-H (Ce). Scale bar = 100 nm.


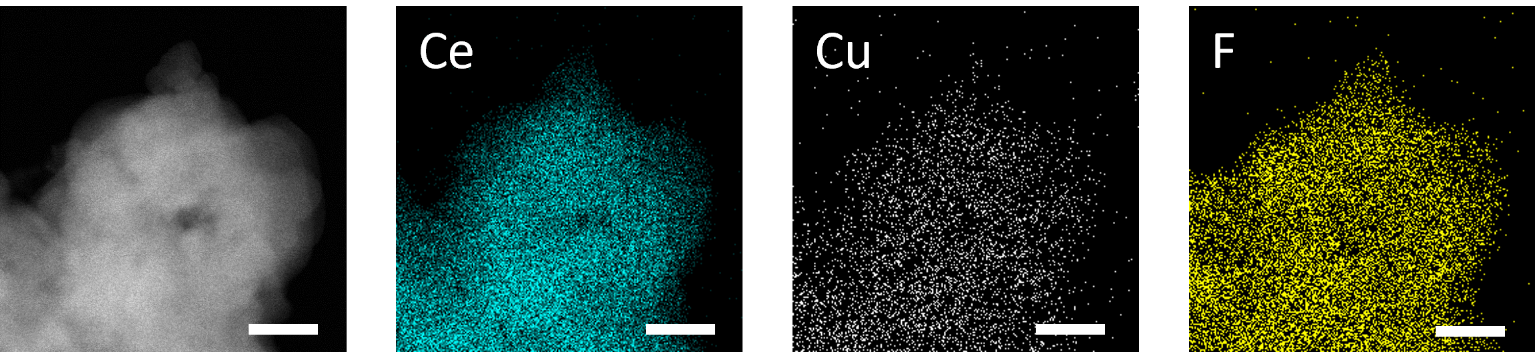


**Figure S9.** HAADF-STEM image and EDX maps of Cu/UiO-66-F (Ce) Scale bar = 100 nm.


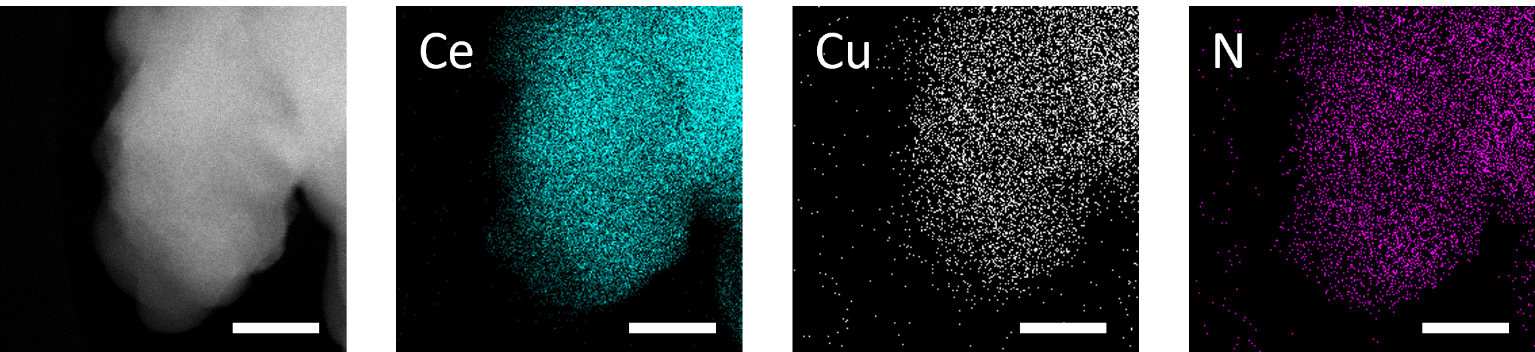


**Figure S10.** HAADF-STEM image and EDX maps of Cu/UiO-66-NH_2_ (Ce). Scale bar = 100 nm.


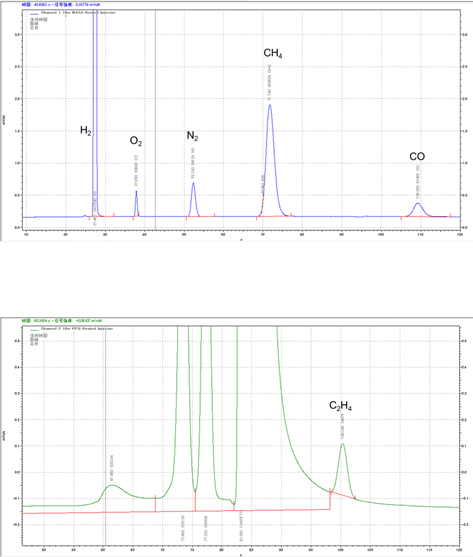


**Figure S11.** Spectra of gas products from Cu/UiO-66-H (Ce) during CO_2_RR at j of 100 mA cm^-2^ detected using GC.


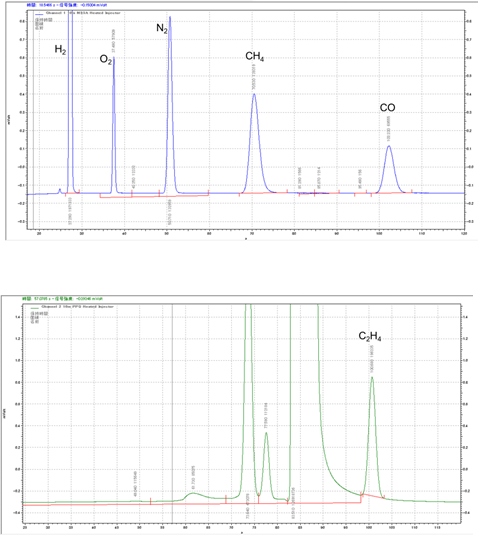


**Figure S12.** Spectra of gas products from Cu/UiO-66-F (Ce) during CO_2_RR at j of 100 mA cm^-2^ detected using GC.


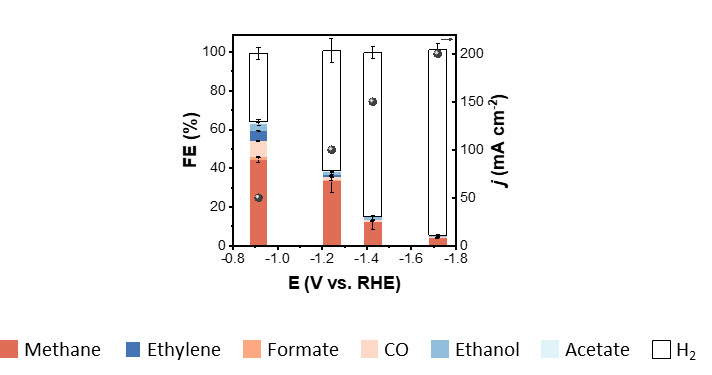


**Figure S13.** CO_2_RR performance of Cu/UiO-66-NH_2_ (Ce).


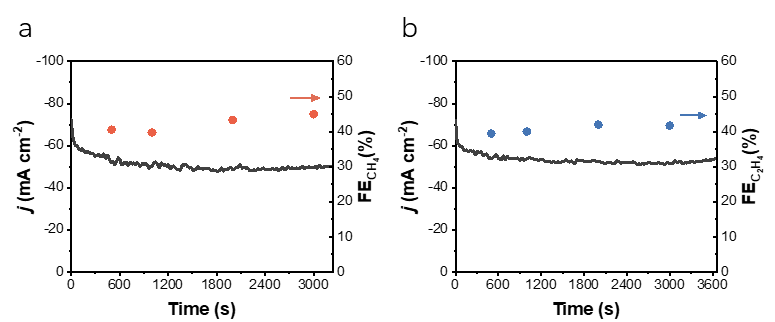


**Figure S14.** Stability measurement of a) Cu/UiO-66-H (Ce) and b) Cu/UiO-66-F (Ce) at -0.8 V vs RHE.


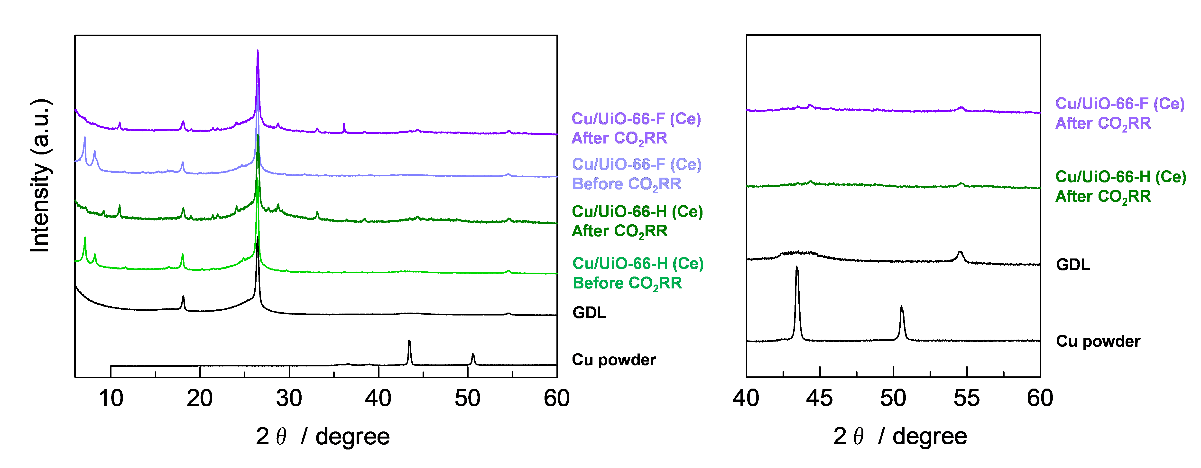


**Figure S15.** XRD patterns of **Cu/UiO-66-H (Ce)** and **Cu/UiO-66-F (Ce)** before and after CO_2_RR.

***
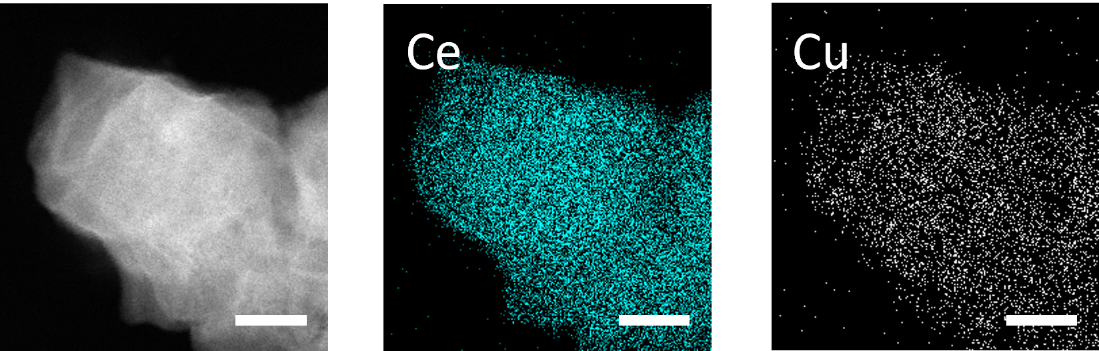
***

**Figure S16.** HAADF-STEM image and EDX maps of Cu/UiO-66-H (Ce) after CO_2_RR. Scale bar = 100 nm.


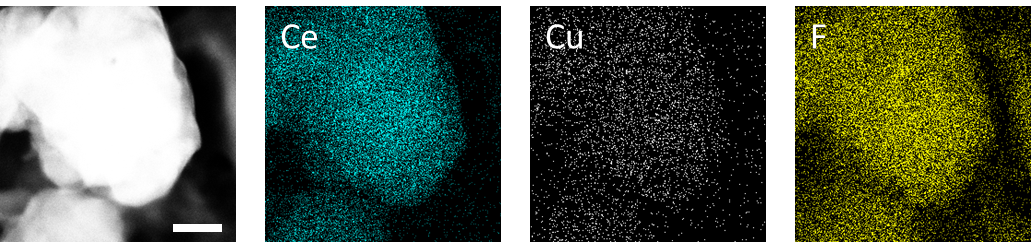


**Figure S17.** HAADF-STEM image and EDX maps of Cu/UiO-66-F (Ce) after CO_2_RR. Scale bar = 100 nm.

# Author Contributions

Conceptualization: M. Sun and J. Cheng

Methodology: M. Sun and J. Cheng

Investigation: J. Cheng, M. Sun, A. Anzai and H. Kobayashi

Visualization: M. Sun and J. Cheng

Funding acquisition: M. Yamauchi

Project administration: H. Kobayashi and M. Yamauchi

Supervision: H. Kobayashi and M. Yamauchi

Writing – original draft: J. Cheng and M. Sun

Writing – review & editing: M. Sun, J. Cheng, A. Anzai, H. Kobayashi and M. Yamauchi

M. Sun and J. Cheng contributed equally to this work.
